# Seasonality Affects Low-Molecular-Weight Organic Acids and Phenolic Compounds’ Composition in Scots Pine Litterfall

**DOI:** 10.3390/plants13101293

**Published:** 2024-05-08

**Authors:** Anna Ilek, Monika Gąsecka, Zuzanna Magdziak, Costas Saitanis, Courtney M. Siegert

**Affiliations:** 1 Department of Botany and Forest Habitats, Faculty of Forestry and Wood Technology, Poznań University of Life Sciences, Wojska Polskiego 71F, 60-625 Poznań, Poland; 2 Department of Chemistry, Faculty of Forestry and Wood Technology, Poznań University of Life Sciences, Wojska Polskiego 75, 60-625 Poznań, Poland; monika.gasecka@up.poznan.pl (M.G.); zuzanna.magdziak@up.poznan.pl (Z.M.); 3 Laboratory of Ecology and Environmental Sciences, Agricultural University of Athens, Iera Odos 75, Votanikos, 11855 Athens, Greece; saitanis@aua.gr; 4 Department of Forestry, Forest and Wildlife Research Center, Mississippi State University, 775 Stone Boulevard, Mississippi State, MS 39762, USA; courtney.siegert@msstate.edule

**Keywords:** *Pinus sylvestris*, soil organic matter, forest soils, organic compounds

## Abstract

Background and Aims: Secondary plant metabolites, including organic acids and phenolic compounds, have a significant impact on the properties of organic matter in soil, influencing its structure and function. How the production of these compounds in foliage that falls to the forest floor as litterfall varies across tree age and seasonality are of considerable interest for advancing our understanding of organic matter dynamics. Methods: Monthly, we collected fallen needles of Scots pine (*Pinus sylvestris* L.) across stands of five different age classes (20, 40, 60, 80, and 100 years) for one year and measured the organic acids and phenolic compounds. Results: Seven low-molecular-weight organic acids and thirteen phenolic compounds were detected in the litterfall. No differences were observed across stand age. Significant seasonal differences were detected. Most compounds peaked during litterfall in the growing season. Succinic acid was the most prevalent organic acid in the litterfall, comprising 78% of total organic acids (351.27 ± 34.27 µg g^− 1^), and was 1.5 to 11.0 times greater in the summer than all other seasons. Sinapic acid was the most prevalent phenolic compound in the litterfall (42.15 µg g^− 1^), representing 11% of the total phenolic compounds, and was 39.8 times greater in spring and summer compared to autumn and winter. Growing season peaks in needle concentrations were observed for all thirteen phenolic compounds and two organic acids (lactic, succinic). Citric acid exhibited a definitive peak in late winter into early spring. Conclusions: Our results highlight the seasonal dynamics of the composition of secondary plant metabolites in litterfall, which is most different at the onset of the growing season. Fresh inputs of litterfall at this time of emerging biological activity likely have seasonal impacts on soil’s organic matter composition as well.

## 1. Introduction

Scots pine (*Pinus sylvestris* L.) is the most widespread species of the *Pinus genus* in the world [1]. It has been widely planted in the United States and occupies a range from Spain in the west to east Russia and from northern Scandinavia to southern Spain [2]. In Poland, Scots pine forests cover most of the country’s forest area (58.2%), constituting the largest share of forests managed by the State Forests (60.3%), while accounting for a slightly smaller proportion in private forests (55.0%) [3]. Scots pine forests comprise over 20% of the productive forest area in Europe, exceeding 28 million hectares [2].

Organic acids and phenolic compounds are secondary metabolites that act as a carbon store while supporting physiological functions in plant metabolism, such as respiration, regulation of stomata, nutrient transport, regulation of plant and soil pH, and interaction with soil [4,5,6]. Secondary metabolites also have a significant impact on the properties of organic matter in soil, influencing its structure and function [7,8].

Phenolic compounds are the most diverse of the secondary metabolites in terms of function and structure. One or several aromatic rings containing one or several hydroxyl groups can be distinguished in their structure. Phenolic compounds play a role in physiological processes related to plant development and extension, including seed germination and cell division [9,10]. Phenolic compounds play a significant role in plants’ resistance to various biotic and abiotic stress and defense mechanisms related to the antioxidative properties (e.g., scavenging and chelating ability) and play a structural function in the cell wall [11,12]. Moreover, for some classes of phenolic compounds, nutraceutical, clinical, antibacterial, and antifungal effects were observed [13,14,15,16].

Concentrations of organic acids have been observed to increase in needles with increasing temperature [6] and in polluted soil environments [17], suggesting their role in needle maintenance and repair from heat stress. However, our understanding of the variability in secondary metabolites in Scots pine needles in natural forest ecosystems is not well documented, nor do we have a clear understanding of the influence of tree age or season on this variation. To address this knowledge gap, we evaluated seven low-molecular-weight organic acids (citric acid, fumaric acid, lactic acid, malic acid, malonic acid, and succinic acid) and thirteen phenolic compounds (2,5-dihydroxybenzoic acid, 4-hydroxybenzoic acid, caffeic acid, catechin, chlorogenic acid, ferulic acid, gallic acid, p-coumaric acid, protocatechuic acid, sinapic acid, syringic acid, t-cinnamic acid, and vanillic acid) in fallen pine needles (*Pinus sylvestris* L.) taken from five pine stands that differed in age. The overall objective of the study was to determine differences in compounds with respect to tree age and the seasonality of sampling. The results can be used to infer potential changes occurring in the biochemistry of organic matter. Organic acids and phenolic compounds contained within organic matter play a significant role in dissolving and mobilizing poorly soluble nutrients in soil (such as Mn, Cu, Zn, Fe, and P) or in metal detoxification (such as Zn and Al), or, as in the case of pedogenesis, they can significantly increase the rate of dissolution of primary minerals and influence weathering [18,19]. The novel research presented within this paper analyzes the seasonal changes in the content of organic compounds in litterfall for the first time. Our results definitively show seasonal variability in the levels of these compounds in litterfall and calls for further research aimed at a better understanding of the influence of litterfall on soil’s organic matter formation and dynamics.

## 2. Results

### 2.1. Organic Acid Contents in Scots Pine Needles

Seven low-molecular-weight organic acids were detected in Scots pine needles. Among them, succinic acid was the most prevalent, comprising more than 78% of the total acid content at 351.27 ± 34.27 µg g^−1^ (Table 1). Lactic, citric, and malic acids comprised 7%, 5%, and 5% of the total acid content found in needles, respectively, while malonic, fumaric, and acetic acids were found in very low concentrations. Stand age was not a significant predictor of the organic acid content in needles (*p* > 0.05, Supplemental Figure S1).

In spring, the most prevalent organic acids that were present in needles were succinic acid (516.0 ± 81.7 μg g^−1^), followed by lactic acid (121.5 ± 33.5 μg g^−1^). Fumaric acid (5.2 ± 0.5 μg g^−1^) and acetic acid (2.0 ± 0.8 μg g^−1^) were observed in the smallest concentrations. Succinic acid was also the most prevalent organic acid in needles sampled during summer (752.5 ± 63.7 μg g^−1^) and autumn (88.8 ± 13.5 μg g^−1^), while acetic acid, lactic acid, and malonic acid were all below detection levels. Citric acid was the most prevalent organic acid in needles sampled in winter (60.6 ± 15.5 μg g^−1^), followed by succinic acid (43.0 ± 10.5 μg g^−1^) and malic acid (31.9 ± 7.2 μg g^−1^). Malonic acid (2.8 ± 1.6 μg g^−1^) and fumaric acid (2.6 ± 0.4 μg g^−1^) were the least common organic acids found in winter needles.

**Figure 1:**
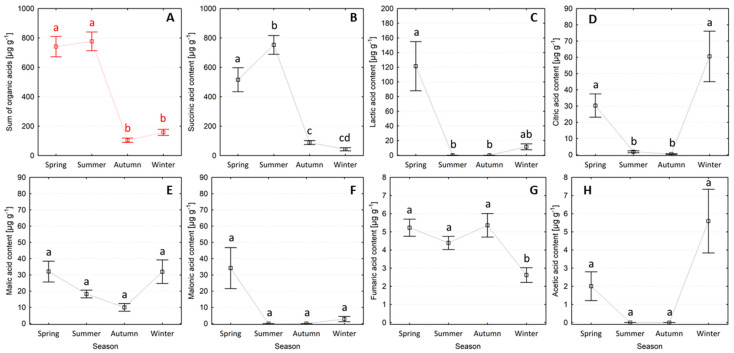
Organic acid content in Scots pine needles collected across four seasons (mean ± SE), where: (**A**) sum of organic acids, (**B**) succinic acid, (**C**) lactic acid, (**D**) citric acid, (**E**) malic acid, (**F**) malonic acid, (**G**) fumaric acid, (**H**) acetic acid. The same letters indicate no statistical differences in the sum of organic acid contents (red color) and individual acids between seasons (Kruskal–Wallis test, *p* < 0.05).

Broadly speaking, needles collected in spring and summer had higher total organic acid contents than needles collected in autumn or winter (Figure 1A), reaching the highest monthly value in July (1287.29 μg g^−1^) (Supplemental Figure S2). The succinic acid content in needles was highest in summer (752.5 ± 63.7 μg g^−1^), which was 1.5 times greater than in spring, and 11.5 times greater than in autumn and winter (Figure 1B). The succinic acid content in needles increased from January (41.84 ± 22.38 μg g^−1^), reaching its maximum in July (1266.30 ± 87.51 μg g^−1^), and then gradually decreased in the following months, reaching the lowest content in December (38.00 ± 9.35 μg g^−1^) (Supplemental Figure S2). The lactic acid needle content was highest in spring (121.47 ± 33.47 μg g^−1^), which was 10.5 times greater than needle concentrations in winter (Figure 1C). Lactic acid was below detection limits in summer and autumn. The highest monthly concentrations of lactic acid were measured in March, April, May, and June (34.50 ± 9.75, 278.97 ± 79.76, 69.11 ± 36.94, and 16.34 ± 8.7 μg g^−1^, respectively) (Supplemental Figure S2C). The citric acid content in needles was similarly high in both the spring and winter seasons (30.28 ± 7.17 μg g^−1^), which was 39.5 times greater than the values observed in summer and autumn (Figure 1D). The largest monthly citric acid content was observed in needles collected in February (113.62 μg g^−1^) (Supplemental Figure S2). The fumaric acid content of needles collected in winter (2.6 ± 0.4 μg g^−1^) was 1.9 times lower than in all other seasons (Figure 1G). The malic acid content exhibited no seasonal trend but was intermittently higher in needles collected during the transitional season months of February and March and September and October (Supplemental Figure S2). The malonic acid and acetic acid needle contents exhibited no seasonal variation (Figure 1E–H).

### 2.2. Phenolic Compound Profiles in Scots Pine Needles

Thirteen phenolic compounds were detected in Scots pine needles. The most dominant phenolic compounds were sinapic acid (42.15 µg g^−1^), gallic acid (36.93 µg g^−1^), and syringic acid (28.01 µg g^−1^), representing 11%, 8%, and 6% of the total phenolic compounds, respectively (Table 1). The mean needle contents of vanillic acid (18.69 ± 2.60), catechin (15.49 ± 1.89), and chlorogenic acid (10.51 ± 0.99) all exceeded 10 µg g^−1^, while all the other phenolic acids were very low, accounting for less than 2% of the total phenolic compounds (Table 1). Stand age was not a significant predictor of the phenolic compound content in needles (*p* > 0.05, Supplemental Figure S2).

All phenolic compounds exhibited some degree of seasonal variation in needle content. In spring, sinapic acid was the most prevalent phenolic compound in needles at 129.4 ± 20.4 µg g^−1^, followed by gallic acid (49.6 ± 4.3 µg g^−1^) and vanillic acid (43.0 ± 9.0 µg g^−1^), while coumaric acid was the rarest phenolic compound (2.8 ± 0.5 µg g^−1^). Sinapic acid (62.1 ± 6.1 µg g^−1^) and gallic acid (48.2 ± 2.7 µg g^−1^) were also the most prevalent compounds in needles sampled in summer, along with syringic acid (57.3 ± 6.9 µg g^−1^). Coumaric acid (8.6 ± 1.1 µg g^−1^), along with 4-hydroxybenzoic acid (7.6 ± 0.9 µg g^−1^), protocatechuic acid (7.5 ± 1.1 µg g^−1^), and ferulic acid (7.4 ± 0.9 µg g^−1^), occurred in the smallest concentrations in summer. Although they were found in smaller concentrations compared to other seasons, gallic acid (23.4 ± 3.5 µg g^−1^) and vanillic acid (10.8 ± 1.3 µg g^−1^) were the most prevalent in autumn-sampled needles, while gallic acid (26.6 ± 6.6 µg g^−1^) and syringic acid (11.5 ± 1.9 µg g^−1^) were the most prevalent in winter-sampled needles. Protocatechuic acid was below detection thresholds across all aged stands in autumn. 2,5-DHBA (0.6 ± 0.2 µg g^−1^), t-cinnamic acid (1.4 ± 0.4 µg g^−1^), and ferulic acid (1.7 ± 0.3 µg g^−1^) exhibited the lowest concentrations in winter needles.

**Figure 2:**
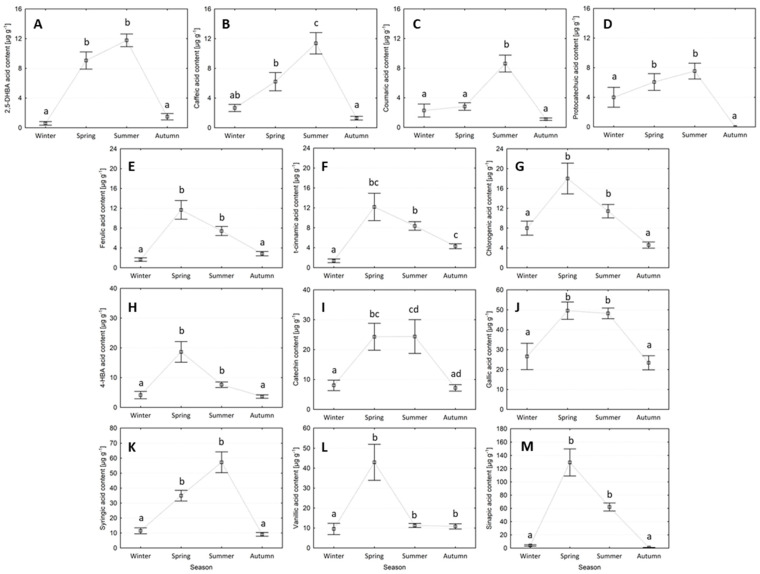
Phenolic compound contents in Scots pine needles collected across four seasons (mean ± SE), where: (**A**) 2,5-DHBA acid content, (**B**) caffeic acid, (**C**) coumaric acid, (**D**) protocatechuic acid, (**E**) ferulic acid, (**F**) t-cinnamic acid, (**G**) chlorogenic acid, (**H**) 4-HBA acid, (**I**) catechin acid, (**J**) gallic acid, (**K**) syringic acid, (**L**) vanillic acid, and (**M**) sinapic acid. The same letters indicate no statistical differences in the individual phenolic compounds between seasons (Kruskal–Wallis test, *p* < 0.05).

Needles sampled in the spring and summer seasons had greater concentrations, while needles sampled in the autumn and winter seasons had lower concentrations. The caffeic and p-coumaric acid contents were all significantly higher in needles sampled in summer compared to all other seasons (Figure 2). The caffeic acid content of needles in summer (2.6 ± 0.5 µg g^−1^) was 1.8 times greater than the caffeic acid in needles sampled in spring and 5.7 times greater than in autumn and winter (Figure 2B). The p-coumaric acid content of needles was 4.2 times greater in the summer (8.6 ± 1.1 µg g^−1^) compared to the other seasons (Figure 2C). The 2,5-DHBA, protocatechuic, ferulic, t-cinnamic, chlorogenic, 4-hydroxybenzoic acid, catechin, gallic, syringic, and sinapic acid contents in needles were all similarly higher in both the spring and summer seasons compared to their lower average values in the autumn and winter seasons (Figure 2). For needles sampled in summer and spring, their acid content of 2,5-DHBA (10.41 ± 1.0 µg g^−1^) was 10.0 times greater, protocatechuic (6.8 ± 1.1 µg g^−1^) was 3.4 times greater, ferulic (9.5 ± 1.4 µg g^−1^) was 4.2 times greater, t-cinnamic (10.3 ± 1.8 µg g^−1^) was 3.6 times greater, chlorogenic (14.7 ± 2.2 µg g^−1^) was 2.3 times greater, 4-hydroxybenzoic acid (13.1 ± 2.2 µg g^−1^) was 3.4 times greater, catechin (24.3 ± 5.1 µg g^−1^) was 3.2 times greater, gallic (48.9 ± 3.5 µg g^−1^) was 2.0 times greater, syringic (46.1 ± 5.3 µg g^−1^) was 4.5 times greater, and sinapic (95.8 ± 13.3 µg g^−1^) was 39.8 times greater compared to needles sampled in autumn and winter. The vanillic acid content of needles was the only phenolic compound that had similarly high levels throughout spring, summer, and autumn (21.7 ± 3.8 µg g^−1^), which were 2.3 times greater than the vanillic acid content in needles sampled during the winter (Figure 2).

**Figure 3:**
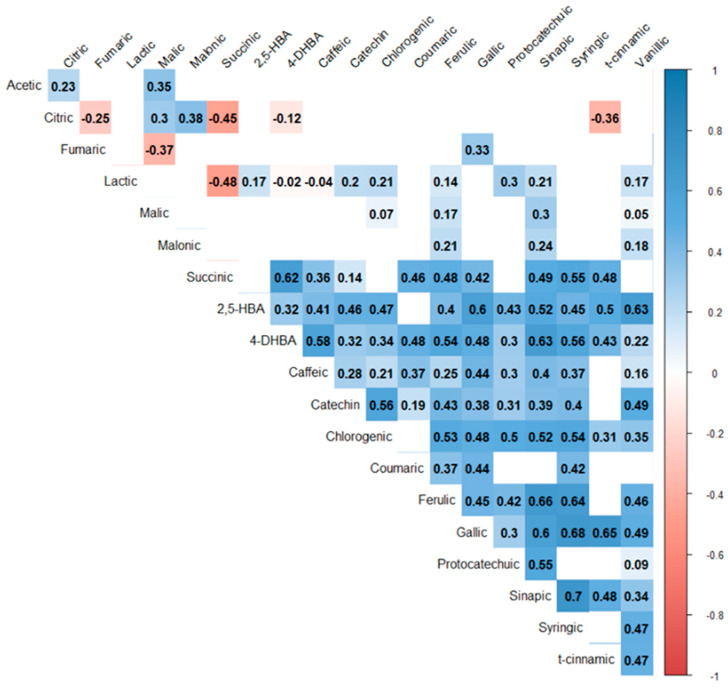
Spearman’s (non-parametric) correlation coefficient (r) matrix of the measured parameters (phenolic compounds and organic acids). Correlation coefficient values are shown in cells where the relationship is significant (*p* < 0.05). Blank cells represent no significant correlation. The corresponding color scale denotes positive (blue-colored tones) and negative (red-colored tones) correlation levels.

Spearman’s (non-parametric) correlation coefficients were calculated and are shown in Figure 3. Correlations were stronger between phenolic compounds compared to organic acids. Within the phenolic compounds, the strongest positive correlations were between sinapic acid and syringic acid (r = 0.70, *p* < 0.001), gallic acid and syringic acid (r = 0.68, *p* < 0.001), and ferulic acid and sinapic acid (r = 0.66, *p* < 0.001). 4-hydroxybenzoic acid was positively correlated with every other phenolic compound, while coumaric acid was only correlated with seven of the twelve other phenolic compounds. Correlations among organic acids and between organic acids and phenolic compounds were less common. Among the organic acids, citric acid and malonic acid had the strongest positive correlation (r = 0.38, *p* < 0.001), followed by acetic acid and malic acid (r = 0.35, *p* < 0.001), citric acid and malic acid (r = 0.30, *p* < 0.001), and acetic acid and citric acid (r = 0.23, *p* < 0.001). There were also negative correlations among organic acids. Succinic acid was negatively correlated with both lactic acid (r = −0.48, *p* = 0.013) and citric acid (r = −0.45, *p* = 0.003). Citric acid was also negatively correlated with fumaric acid (r = −0.25, *p* = 0.004), and fumaric acid was negatively correlated with malic acid (r = −0.37, *p* < 0.001). Lactic acid was correlated, both positively and negatively, with nine phenolic compounds, exhibiting the strongest correlations with protocatechuic acid (r = 0.30, *p* < 0.001). Fumaric acid exhibited the strongest positive correlation with the phenolic acid gallic acid (r = 0.33, *p* < 0.001). Citric acid exhibited the strongest negative correlation with the phenolic acid t-cinnamic (r = −0.36, *p* = 0.022). Acetic acid was the only organic acid that was not correlated with any phenolic compounds.

## 3. Discussion

In this study, we evaluated differences in organic acid and phenolic compounds in Scots pine needle leaves collected monthly across stands of different ages. We found that both organic acid and phenolic compounds in pine needles varied monthly and seasonally but did not vary across stand ages. Summed together, the organic acid needle content was highest in spring and summer and declined in autumn and winter, which was largely driven by succinic acid. In contrast, the citric acid formation was highest in winter and spring. The temporal trends of phenolic compounds were similar, with peak values during growing season months. However, phenolic compounds occurred more evenly, with several compounds (sinapic acid, gallic acid, syringic acid, and vanillic acid) occurring in near-equal proportions.

The physiological functions of plants, including respiration, osmotic regulation, transport of nutrients, and pH regulation in plant cells and the rhizosphere, require the presence of carbon compounds in the form of sugars, organic acids, phenolic compounds, and amino acids [4]. These organic acids are essential molecules, secreted by plant roots and taking part in processes such as the detoxification of heavy metals, adaptation to the environment, and facilitation of nutrient uptake. The essential physiological functions of organic acids ensure a redox balance, support ionic gradients on membranes, and acidify the extracellular environment [4]. Furthermore, the accumulation, biosynthesis, transport, and secretion of organic acids in the rhizosphere increase in response to environmental stress [20]. The most frequently occurring acids in plants (roots, bark, leaves/needles) are citric, malic, succinic, and fumaric acids. Citric acid, depending on the intensity of reactions related to the conversion of this compound [4], is necessary for the acquisition and transport of iron in the plant [21].

Organic acids play an important role in other plant organs, where they are produced in mitochondria and stored in vacuoles [20,22,23]. Here, these molecules are involved in the processes of anabolic and catabolic metabolic pathways, which are necessary for primary metabolism, stomatal regulation, and the storage of bound carbon compounds [5,24,25]. Succinic acid has a powerful antioxidant effect [26] and is essential to the citric acid cycle and helps produce respiration energy [27,28]. Interestingly, we found a strong negative relationship between succinic and citric acids, which may imply that they are produced in the greatest concentrations in opposite seasons, alternating when each is near its peak. Malic acid is involved in the transfer of redox equivalents between cellular compartments, playing the role of osmolyte and anion in compensating for the positive charge of potassium, which is particularly important in stomatal responses [21,29,30]. Citric, succinic, and fumaric acids are the main components of the tricarboxylic acid pathway. Lactic acid is a signaling molecule, a precursor of glucose production, and a source of energy for mitochondria [31]. It has also been shown that lactic acid fermentation under the influence of bacteria significantly affects plants’ total content of phenols. However, the biochemical production of these compounds depends on plant species, environmental conditions (e.g., drought, presence of metals), stage of plant development, and age [24]. As such, our understanding of these relationships, especially in field settings, is lacking and requires further investigation.

Our research identified differences in the seasonal variability of individual organic acids in needles of Scots pine at a compound-specific level. Ref. [32] showed that malic acid has a clear seasonal tendency, with concentrations being low in summer and high in winter, as was observed in our study. In turn, Ref. [25] observed citric and malic acids decreasing from June to October, at which point the lowest concentrations of these acids were observed. We also observed a decline in citric acid throughout the growing season. The general increase in citric and malic acid during winter provides equivalent reductions [33] and is associated with increased metabolic activity at the onset of the growing season [6]. Citrate can also be used as a carbon-transporting metabolite, transporting carbon to sinks such as roots and facilitating the transport of nutrients such as iron towards the needles [4]. Confirmed relationships, where seasonal variations in the content of organic acids (especially malic and citric acids) are observed, suggest increased investment in maintenance and repair mechanisms. Therefore, the seasonal cycle of organic acids can be considered an indicator of modified carbon metabolism in leaves and possibly in other tree tissues [6,25]. Succinic acid and fumaric acid were the only two organic acids that peaked in the summer months in our study. Succinic acid primarily normalizes cell metabolism and supports the formation of new ones, so the observed increase aligns temporally with when the elongated needle process was the most intense. The observed variability of organic acids in the needle litter of Scots pine appears to stem from environmental conditions and seasonal fluctuations. It is suggested that this variability may consequently have a significant impact on the nature of organic matter. Therefore, the presented results provide a robust basis for further research aimed at understanding how the observed variability in organic acid content may influence the chemistry and dynamics of organic matter.

Phenolic compounds in needles have also been documented in other studies evaluating Scots pine [34,35,36]. The composition of phenolic compounds in trees is related to genetic and environmental parameters, including species, age, tissue type, and environmental conditions [13,37,38,39,40,41]. Ref. [42] identified different classes of phenolic compounds in four species of *Pinus* (*P. peuce*, *P. nigra*, *P. mugo,* and *P. sylvestris*), including flavonoid glycosides, phenolic acids, and procyanidins. In *P. halepensis* needles, the dominant phenolic compound was protocatechuic acid, which was only observed in very low concentrations in our study’s needles. The authors of [34] noted that catechin, phenolic glycosides, and their derivatives were the main compounds in different tissues of Scots pine.

Exposure to UV-B radiation generates reactive oxygen species, which affect DNA and damage protein. In response, plants synthesize phenolic compounds to protect their tissue and regulate the antioxidant mechanisms at the cellular and whole-organism levels [43,44,45]. In Poland, where our study was conducted, UV radiation is the lowest from November to March (autumn, winter). The sharp increase in most of the phenolic compounds in needles coincided with the increase in UV-B radiation and the onset of the growing season. During summer, the synthesis of phenols stabilized, and it decreased in the autumn and winter. Considering a more detailed analysis of seasonal changes, the spring surge of most of the compounds analyzed was followed by a decrease in their content and another spike. Such changes may result from changes in the amount of radiation and periods of drought in the summer. The production of phenolic acids to repair cell damage from air pollutants (ozone, sulfur dioxide, nitrogen dioxide) during the summer months may also occur [46]. Additionally, catechin has been shown to increase under elevated CO_2_ treatments, where extra carbon from enhanced photosynthesis is available for the production of secondary metabolites [47]. Phenolic acids also have strong antibacterial and antimicrobial activity [48,49,50,51]. Ferulic, protocatechuic, and coumaric acids have antifungal activity that inhibits pathogen growth during the growing season [52,53]. In total, we did not observe direct relationships between phenolic compounds and tree age; instead, our results suggest that Scots pine produce compounds in response to seasonal physiology and environmental stressors more broadly.

We observed strong correlations between many of the phenolic compounds that were not present among the organic acids. This is likely due to shared biosynthesis pathways, in which the compounds are produced. For example, the strong correlation that was found between sinapic and syringic acids may be attributed to the fact that they are derivatives of the same phenylpropanoid pathway, which involves the conversion of phenylalanine into cinnamic acid, which in turn is converted into sinapic and syringic acids [54]. Furthermore, the correlations between phenolic compounds and organic acids are likely related to their shared response mechanisms and their roles in stress-related defenses. Many studies have shown that certain phenolic compounds, especially those that are exuded by roots, can influence soil’s microbial activity and population composition, because they serve as both microbial substrates and toxins in the soil [55]. On the other hand, it has been observed that the phenolic composition and properties of humic substances in soils depend on the chemical composition, including the phenolic profile, of inputs from decomposing plant material [56,57,58]. Therefore, it is worthwhile to continue these studies to examine the impact of phenolic compounds originating from fallen Scots Pine needles on the composition of organic matter and microbial communities in forest soils and, consequently, on soil quality.

The results of this study provide insights into the impact of variability in the content of organic acids and phenols in needle litterfall on changes in organic matter and their potential further influence. Particularly noteworthy is the observation of high concentrations of these compounds in spring and summer, regardless of tree age. Further research is needed to better understand how these seasonally variable components affect soil processes and how they may further shape the dynamics of organic matter. Additionally, studies covering a wider geographical range are needed to determine the full extent of these changes and their potential consequences for forest ecosystems.

## 4. Materials and Methods

### 4.1. Study Site and Litterfall Sampling

Litterfall samples were collected in Scots pine stands in the Czarnobór Forest District in northwestern Poland during a field campaign reported in [59]. Samples were collected across five forests, differing in age (~20, 40, 60, 80, and 100 years old). Within each forest stand, one 100-m-long transect was delineated, and five litterfall traps were placed equidistant along each transect (25 litterfall traps in total). Pine needles were collected from the litterfall traps monthly for one year (12 sampling periods) in each stand, from April 2019 to March 2020. Pine needles were stored on ice and transported back to the lab, where they were stored at −14 °C until chemical analyses were conducted. The characteristics of the Scots pine stands, their location, and information on the sampling date of the needles have been described in detail in [59].

### 4.2. Extraction of Organic Acids and Phenolic Compounds from Scots Pine Needles

Litterfall samples were prepared for analysis of organic acids and phenolic compounds. For each monthly sample collection, approximately 2.5 g of needles were ground to a powder in a mortar that was chilled using liquid nitrogen. Ground samples were collected in 50 mL centrifuge tubes and stored at −80 °C until analysis according to the modified method of [60,61]. For organic acid analysis, 5 mL of H_2_O was added to the ground litterfall sample, and then, the mixture was heated in a water bath (60 min, 80 °C) to denature the degradative enzymes. For phenolic compound analysis, the ground litterfall samples were mixed with 80% methanol and HCl (99:1). Samples were sonicated and shaken for 5 h at 40 °C in a water bath (Bandelin Sonorex DL 102 H, Germany) and then shaken in orbital shakers for 8 h (Ika KS 260 shaker, IKA-Werke GmbH & Co. Kg, Staufen, Germany). Aqueous and methanolic solutions of needle samples were centrifuged at 3600 rpm/min for 15 min at 25 °C (Universal 320R Hettich Zentrifugen, Tuttlingen, Germany) and then filtered through 0.2 μm nylon filters.

### 4.3. Chromatographic Analysis

Organic acids and phenolic compounds were analyzed using a Waters Acquity H-class Ultra Performance Liquid Chromatography (UPLC) system (Waters Corporation, Milford, MA, USA). Separation was achieved on a thermostated 35 °C column Acquity UPLC BEH C18 (150 mm × 2.1 mm, 1.7 µm, Waters, Milford, CT, USA). Elution was performed with the mobile phase composed of A (water, containing 0.10% formic acid) and B (acetonitrile, containing 0.10% formic acid) at a flow rate of 0.4 mL/min. Detection was carried out at λ = 280 nm as the preferred wavelength for the following organic acids: acetic, citric, fumaric, lactic, malic, maleic, malonic, oxalic, quinic, and succinic. It was carried out at the same wavelength for the following phenolic compounds: gallic acid, protocatechuic acid, 4-hydroxybenzoic acid, vanillic acid, catechin, syringic acid, and t-cinnamic acid. Detection was carried out in a Waters Photodiode Array Detector (Waters Corporation, Milford, MA, USA) at λ = 320 nm for the following phenolic compounds: caffeic acid, chlorogenic acid, sinapic acid, 2,5-dihydroxybenzoic acid, p-coumaric acid, and ferulic acid. Then, 5 μL of liquor was injected into a UPLC BEH C18 column to determine the organic compounds. Compounds were identified by comparing the retention time of the peaks with the retention time of standards or by adding a specific amount of the standard to the analyzed samples and repeated analysis. The results were expressed in micrograms per gram of fresh weight (f.w.) of needle samples [μg g^−1^ f.w.].

### 4.4. Statistical Analysis

Statistical analysis and associated graphics were performed in Statistica 13.3 PL software (StatSoft Inc., Tulsa, OK, USA). Data were checked for normality using the Shapiro–Wilk test and were found to be non-parametric. Therefore, we used Kruskal–Wallis and Dunn’s post hoc tests to identify significant differences in compound contents between stand age, sampling month, and season. Seasons were delineated following [59], where January–March are winter, April–June are spring, July–September are summer, and October–December are autumn. All tests were performed at a significance level of α = 0.05. Moreover, Spearman’s (non-parametric) correlation coefficients were computed for all measured compounds using the function “cor” in the “stats” package in R [62] and plotted using the function “corrplot” in the “corrplot” package [63]. All R analysis was carried out in version 4.2.2.

## 5. Conclusions

Our study offers valuable insights into the seasonal dynamics of organic acids and phenolic compounds within Scots pine litterfall across stands of varying ages. We found distinctive variations in organic acid and phenolic compound concentrations over time but did not observe differences across stand ages. The seasonal patterns revealed higher levels of organic acids, notably, succinic and fumaric acids, during the summer months, coinciding with increased metabolic activity and needle elongation. Conversely, citric and malic acids showed a peak in the winter months and declined during the growing season, potentially indicating altered carbon metabolism and increased investment in maintenance and repair mechanisms. Furthermore, our investigation highlighted changes in phenolic compounds throughout the seasons, likely in response to UV-B radiation and the onset of the growing season. These compounds, known for their antioxidant properties, serve as protective agents against environmental stressors and potentially harmful air pollutants. As such, trees’ physiological properties are reflected in their litterfall, which serves as input to the forest floor.

While our study contributes to comprehending the secondary metabolite dynamics in this common European tree species, further research across diverse geographical locations is necessary to develop a comprehensive understanding of the trends observed in organic acid and phenolic compound production. Such future investigations can enhance our understanding of these compounds’ potential applications and their role in environmental adaptation and stress response in trees.

## Figures and Tables

**Table 1:** Mean and standard deviation of organic acids and phenolic compounds in Scots pine needle samples. Values were computed across all ages of forest stands and across all months.

Organic Acid	Mean Content (µg g^−1^)	Phenolic Compound	Mean Content (μg g^−1^)
Succinic	351.27 ± 34.27	Sinapic Acid	49.18 ± 6.58
Lactic	33.28 ± 9.12	Gallic Acid	36.98 ± 2.42
Citric	23.51 ± 4.62	Syringic Acid	28.06 ± 4.50
Malic	23.06 ± 2.63	Vanillic Acid	18.69 ± 2.60
Malonic	9.30 ± 3.33	Catechin	15.49 ± 1.89
Fumaric	4.40 ± 0.25	Chlorogenic Acid	10.51 ± 0.99
Acetic	2.24 ± 0.50	4-hydroxybenzoic acid	8.64 ± 1.05
Total	447.07 ± 33.82	t-Cinnamic Acid	6.66 ± 0.79
		2,5-dihydroxybenzoic acid	5.94 ± 0.51
		Ferulic Acid	5.88 ± 0.61
		Caffeic Acid	5.24 ± 0.56
		Protocatechuic Acid	4.68 ± 0.54
		p-Coumaric Acid	3.89 ± 0.43
		Total	447.07 ± 33.82

## Data Availability

The data will be made available by the authors on request.

## Supplementary Materials

The following supporting information can be downloaded at https://www.mdpi.com/article/10.3390/plants13101293/s1: Figure S1: Organic acid content in *P. sylvestris* needles as a function of stand age (mean ± SE). Significant differences (*p* < 0.05) between stand ages as computed from non-parametric Kruskal-Wallis test are denoted with different lowercase letters.; Figure S2: Organic acid content in *P. sylvestris* needles as a function of sampling month (mean ± SE). Significant differences (*p* < 0.05) between sampling months as computed from non-parametric Kruskal-Wallis test are denoted with different lowercase letters.
