# Supplementary material for: Seasonality Affects Low-Molecular-Weight Organic Acids and Phenolic Compounds’ Composition in Scots Pine Litterfall
Source: Plants (Basel). 2024 May 8;13(10):1293. doi: 10.3390/plants13101293 (PMC11125096; doi:10.3390/plants13101293)
Supplement: Supplementary file 1 [file plants-13-01293-s001.zip › plants-2997676-supplementary.pdf]

Seasonality Affects Low-Molecular-Weight Organic Acids and Phenolic Compounds' Composition in Scots Pine Litterfall

Anna Ilek [1,*], Monika Gąsecka [2], Zuzanna Magdziak [2], Costas Saitanis [3], and Courtney M. Siegert [4]

1. Department of Botany and Forest Habitats, Faculty of Forestry and Wood Technology, Poznań University of Life Sciences, Wojska Polskiego 71F, 60-625, Poznań, Poland; anna.ilek@up.poznan.pl
2. Department of Chemistry, Faculty of Forestry and Wood Technology, Poznań University of Life Sciences, Wojska Polskiego 75, 60-625 Poznań, Poland; monika.gasecka@up.poznan.pl, zuzanna.magdziak@up.poznan.pl
3. Laboratory of Ecology and Environmental Sciences, Agricultural University of Athens Iera Odos 75, Votanikos, 11855, Athens, Greece; saitanis@aua.gr
4. Department of Forestry, Forest and Wildlife Research Center, Mississippi State University, 775 Stone Boulevard, Mississippi State, MS 39762, USA; courtney.siegert@msstate.edu

* Correspondence: anna.ilek@up.poznan.pl

Supplemental Information

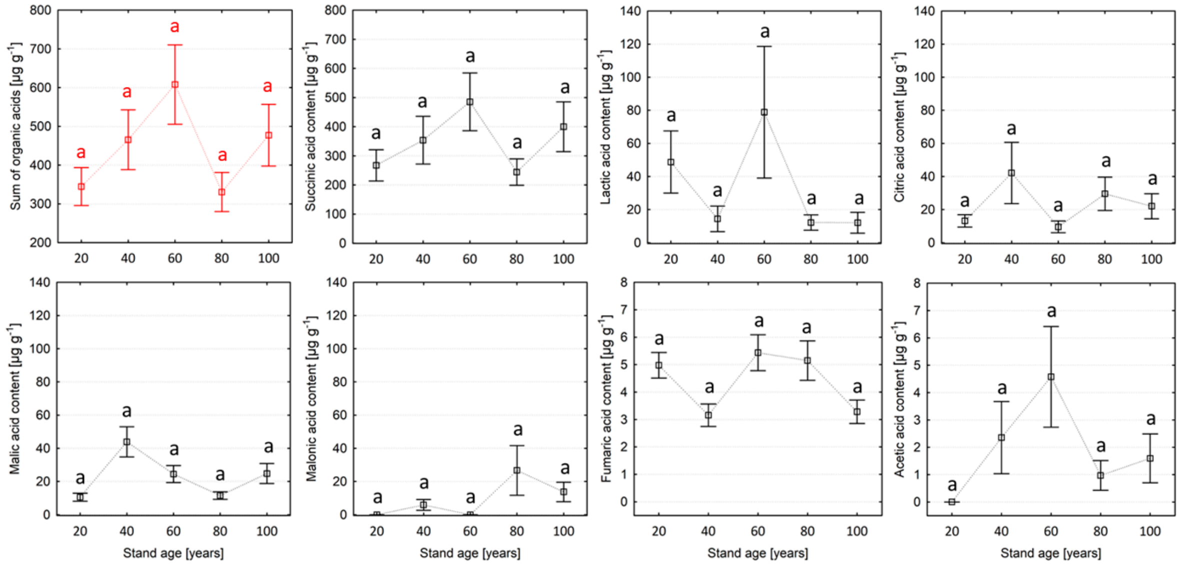

Figure S1. Organic acid content in *P. sylvestris* needles as a function of stand age (mean ± SE). Significant differences ($p < 0.05$) between stand ages as computed from non-parametric Kruskal-Wallis test are denoted with different lowercase letters.

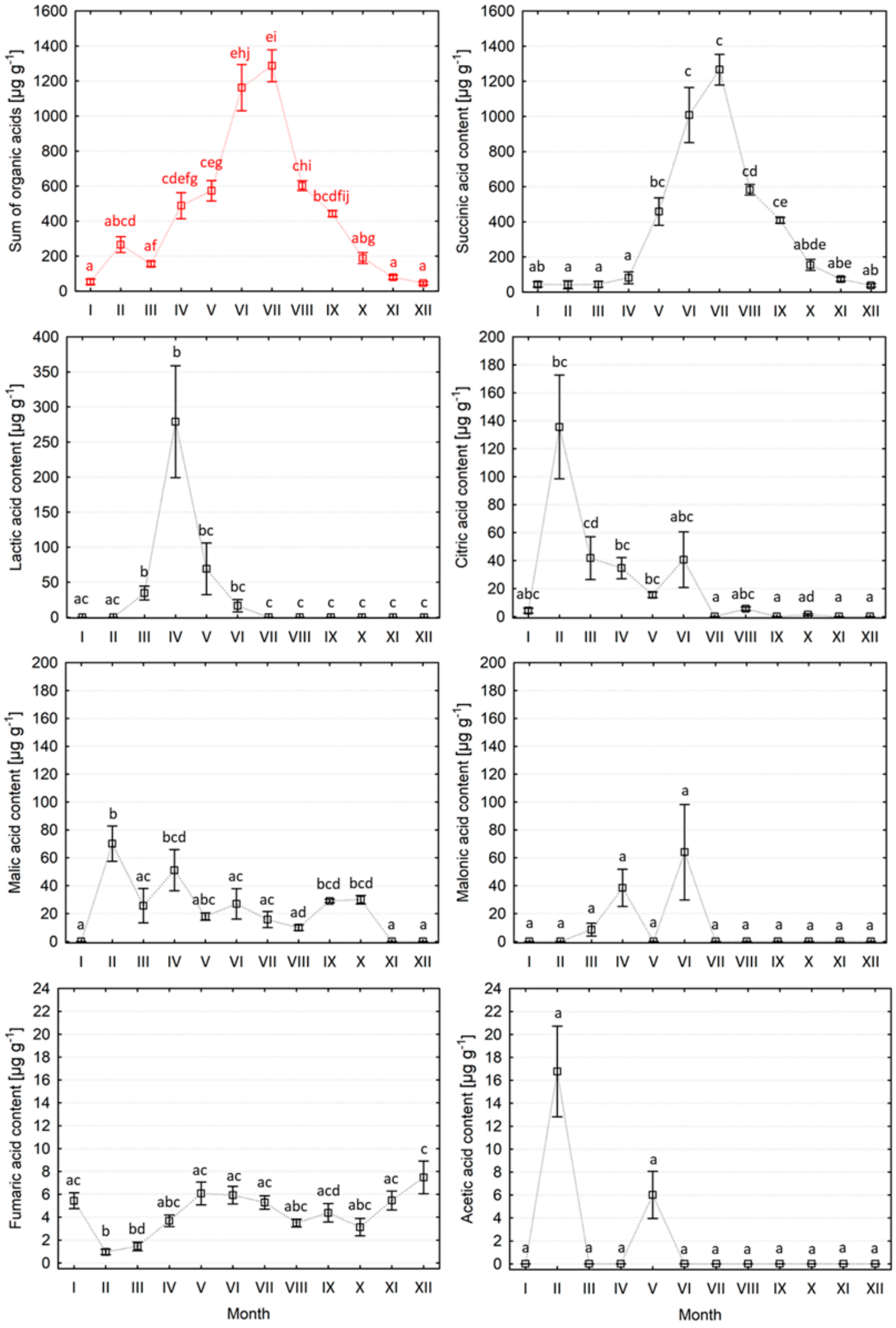

Figure S2. Organic acid content in *P. sylvestris* needles as a function of sampling month (mean ± SE). Significant differences ($p < 0.05$) between sampling months as computed from non-parametric Kruskal-Wallis test are denoted with different lowercase letters.
